# Validation of analytical method for rhynchophorol quantification and stability in inorganic matrix for the controlled release of this pheromone

**DOI:** 10.1186/s13065-018-0426-1

**Published:** 2018-05-10

**Authors:** Arão Cardoso Viana, Ingrid Graça Ramos, Edeilza Lopes dos Santos, Artur José Santos Mascarenhas, Marcos dos Santos Lima, Antônio Euzébio Goulart Sant’Ana, Janice Izabel Druzian

**Affiliations:** 1 Faculty of Pharmacy/RENORBIO, Federal University of Bahia, Rua Barão de Jeremoabo, 147, Campus Universitário de Ondina, Salvador, BA 40170-115 Brazil; 2 Department of Food Technology, Federal Institute of Sertão Pernambucano, Campus Petrolina, BR 407, Km 08, Jardim São Paulo, Petrolina, PE 56314-520 Brazil; 3 Faculty of Pharmacy, Federal University of Bahia, Rua Barão de Jeremoabo, 147, Campus Universitário de Ondina, Salvador, BA 40170-115 Brazil; 4 Department of Chemistry, Federal Institute of Bahia, Rua Emídio dos Santos, s/n, Barbalho, Salvador, BA 40301-015 Brazil; 5 Institute of Chemistry, Federal University of Bahia, Rua Barão de Jeremoabo, 147, Campus Universitário de Ondina, Salvador, BA 40170-115 Brazil; 6 Center of Agricultural Sciences, Federal University of Alagoas, Av. Lourival Melo Mota s/n, Campus A. C. Simões, Maceió, AL 57072-900 Brazil

**Keywords:** Semiochemical, Zeolite, Clay, Controlled release, *Rhynchophorus palmarum* L.

## Abstract

A fast method for the identification and stability evaluation of the aggregation pheromone rhynchophorol, which is the main substance used for chemical communication by the beetle *Rhynchophorus palmarum* L., was validated. In addition, the technique was applied to the evaluation of two inorganic matrices, with the objective of using them as controlled-release devices. The analytical method showed good linearity (R^2^ = 0.9978), precision (CV% < 1.79), recovery (84–105%) and limits of detection (0.2 mg mL^−1^) and quantification (0.3 mg mL^−1^); in compliance with the validation legislation established by ANVISA. In the interaction study, the inorganic matrices zeolite L and Na-magadiite showed high rates of pheromone recovery without promoting its degradation for a period of 180 days, which is not reported in the literature for other matrices. The structures of the zeolite L/rhynchophorol and Na-magadiite/rhynchophorol composites showed slower release kinetics during the storage period when compared with pure pheromone, which is desirable since it extends the period of rhynchophorol release and decreases the negative effects caused by the environmental parameters. 
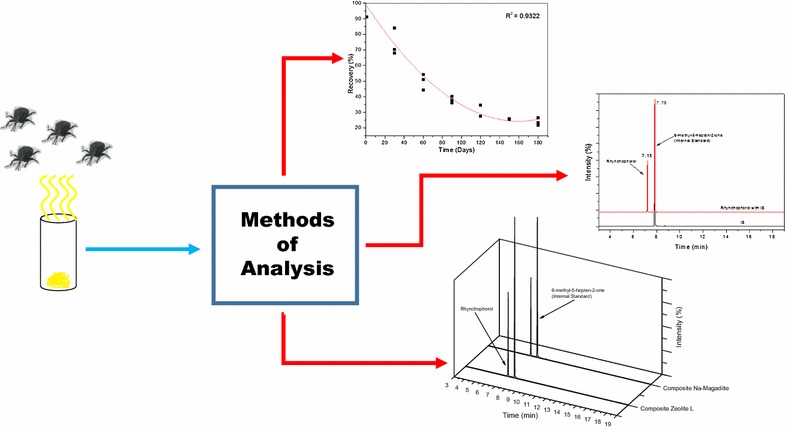

## Introduction

The beetle *Rhynchophorus palmarum* L. is an insect of the family Dryophthoridae, subfamily Rhynchophorina and class Rhynchophorini [1].

This insect is a recurrent pest, which attacks mainly sugarcane (*Saccharum officinarum*) and coconut (*Cocos nucifera* L.) plantations, damaging the stalks of these plants in the search for food and reproduction sites, and laying eggs which will later hatch [2]. However, the highest risk posed by this beetle is its use as a vector by the nematode *Bursaphelenchus cocophilus.* This nematode is the main agent responsible for causing the disease in coconut trees known as red ring, which rapidly leads to the death of the plant. In order to control the populations of this nematode, the main strategy is to eliminate the insect *Rhynchophorus* L. and its larvae, so that the number of individuals is maintained at acceptable levels and the economic viability of coconut cultivation is preserved [3].

The aggregation pheromone 6-methyl-2-hepten-4-*ol* (rhynchophorol), released by *R. palmarum* L. at the time of feeding to attract other individuals and also promote reproduction, has been used as an alternative for the control of this pest, due to its potential use together with biological traps [3, 4]. The control of *Rhynchophorus ferrugineus*, an insect of the same genus as *R. palmarum* L., can be carried out using natural enemies such as viruses, bacteria, fungi, yeasts, nematodes and mites, of which the use of fungi is the most common. However, the use of these natural enemies is not effective against all insects of the *Rhynchophorus* genus, since the success of this strategy is influenced by the insect dispersion and environmental variations [5].

Some materials have been studied for the controlled release of pheromones, including zeolites, nanoencapsulates and nanosensors [6]. The choice of the adsorbent matrix must be made cautiously, aimed at guaranteeing the maximum efficiency of the composite formed (matrix/pheromone) without contributing to the degradation of the pheromone during its preparation or storage [7]. In the selection process, some characteristics of the matrix should be observed, such as: pheromone release kinetics as close to zero as possible, low production cost and maintenance of pheromone stability.

Some structures for the pheromone controlled release matrix have been studied, such as: sepiolite clay [8]; whey protein with acacia gum for microencapsulation [9]; plastic pipette tips [10]; zeolites ZSM-5, silicalite-1, faujasite and beta zeolite [7, 11]. The use of pheromone rhynchophorol together with mass-traps has been studied and implemented over the years, seeking to improve the efficiency of the application of this technique and enable the capture of the highest number of insects during the period of control [12–14].

The aims of this study were to validate an analytical method for the identification and quantification of the aggregation pheromone rhynchophorol and to develop a composite comprised of an inorganic matrix and rhynchophorol for the chemical attraction of the beetle *R. palmarum* L. A controlled release study was carried out and the interaction of the pheromone with the Na-magadiite and zeolite L matrices was investigated.

## Materials and methods

### Chemicals

The rhynchophorol (2-methyl-5(E)heptenone-4-*ol*) standard, with a purity greater than 99%, was donated by Interacta Química Ltd (Alagoas, Brazil). HPLC-grade *n*-hexane (Mallinckrodt ChromAr) was used as the organic solvent. The substance 6-methyl-5-hepten-2-one with 99% purity (Sigma-Aldrich) was used as the internal standard.

As starting reagents for the Na-magadiite synthesis, the following materials were used: NaOH (Synth), hexamethyleneimine (HMI, Sigma-Aldrich, 99%), Aerosil 200 silica (Degussa) and NaCl (Sigma-Aldrich).

### Inorganic structures

The Na-magadiite lamellar structure was obtained through the synthesis method proposed by Elyassi et al. [15] for the obtainment of zeolite ITQ-1 with modifications. In this synthesis, the hydrothermal process was carried out in the static form over a period of 7 days. The gel formed was described as: SiO_2_: 0.31HMI:0.15NaCl:0.31NaOH:44H_2_O. In addition, the TMAdaOH was replaced by NaOH.

### Characterization of the samples of the inorganic matrices

X-ray diffraction (XRD) was carried out with a Shimadzu diffractometer (model XRD6000), with CuKα radiation at 40 kV and 30 mA, carrying out the reading from 5° up to 55° 2θ at a velocity of 2° min^−1^. The identification of the clay composition was performed with the aid of an energy dispersive X-ray (EDX) spectrometer (Shimadzu EDX-720) with a rhodium radiation source, operating at 15 kV (Na to Sc) or 50 kV (Ti to U) with a collimating slit of 10 mm [7].

### Methodology for the determination of rhynchophorol by CG-MS

Prior to performing the analytical method, the best evaluation parameters were sought in order to aid the identification, separation and quantification of the pheromone and the internal standard with the equipment used. Conditions for the heating rate of the ramp, injection temperatures, flow velocities, and analysis time were optimized.

This analytical method validation was based on the category II classification of the Guide for Validation of Analytical and Bioanalytical Methods of ANVISA, aimed at quantitative or limit tests for the determination of the impurities and degradation products in pharmaceutical products and raw materials [16]. The parameters of linearity, specificity, recovery, precision, detection limit and quantification limit were evaluated.

#### Linearity and specificity

Seven concentrations of the pheromone rhynchophorol, varying from 0.86 up to 43 mg mL^−1^, were prepared in triplicate. The samples were diluted in 1 mL of HPLC-grade *n*-hexane together with 10 µL of 6-methyl-5-hepten-2-one. An internal standard (IS) was used. The areas for each substance were obtained through the peak integration with the aid of the TurboMass software program (version 5.4.21617), along with the retention time. The analytical curve for the correlation between the rhynchophorol/IS areas was constructed.

#### Recovery and precision

In order to evaluate the rhynchophorol recovery, triplicate samples containing 10 µL of pheromone were adsorbed onto 50 mg of the zeolite L inorganic structure. The system was shaken for 1 min. After being left to stand for 4 h at ambient temperature, 2 mL of *n*-hexane was added to the system followed by shaking for 1 min. The system was then left to stand for 4 h. After this period, the system was shaken again for 1 min and left to stand 1 min again, where the supernatant was removed and filtered through a nylon membrane of 0.45 µm (Allcrom/Brazil). The supernatant was later analyzed by GC–MS.

#### Detection limit (DL) and quantification limit (QL)

In order to determine the DL and QL values, samples of the pheromone rhynchophorol were prepared and evaluated with the aid of the signal-to-noise ratio tool, provided in the TurboMass software program (version 5.4.2.1617), installed in the equipment used. To obtain the DL and QL values, signal-to-noise ratios of 2:1 and 10:1, respectively, were considered as established by ANVISA [16, 17].

### Preparation of composites of inorganic matrix and rhynchophorol

Composites were formed through the interaction of the inorganic matrices used in this study with the pheromone rhynchophorol. The methodology described by Ramos et al. [7] was applied in the preparation procedure. 50 mg of the lamellar structured Na-magadiite or zeolite L was placed in an Eppendorf^®^ Safe-Lock tube (2 mL/polypropylene) and 10 µL (~ 8.1 mg) of rhynchophorol was added. The system was shaken for 1 min and later kept under storage at room temperature (20–25 °C) for 24 h.

### Evaluation of the stability of composites

The stability of the pheromone adsorbed onto the matrix was evaluated through the extraction and recovery of the adsorbed rhynchophorol according to the procedure describe in “Preparation of composites of inorganic Matrix and rhynchophorol”. The samples were placed in sealed Eppendorf^®^ Safe-Lock tubes (2 mL/polypropylene) and kept in a temperature controlled (25 °C), without forced ventilation and protected from light. Quintuplicate samples were prepared and the extraction was carried out over a period of 1–180 days, with intervals of 30 days between each analysis.

In this procedure, 2 mL of *n*-hexane was added to the system, which was shaken for 1 min and then left to stand for 4 h. After this period, the system was shaken again for 1 min and the supernatant was removal and filtered through a nylon membrane of 0.45 µm (Allcrom/Brazil).

### Quantification of the recovered rhynchophorol by CG-MS

The amount of rhynchophorol recovered was determine using a gas chromatograph (Clarus 680), coupled to a mass spectrometer detector (Clarus 600C), with an ELITE-5MS capillary column (Perkin Elmer/USA). Samples (1 µL) were injected through a CTC Combipal automatic injector (Pal System/Switzerland). The run conditions were: helium carrier gas with 1 mL min^−1^ flow, 50 mL split, and injector temperature of 150 °C. The initial temperature of the oven was 50 °C for 3 min with a heat ramp of 10 °C min^−1^ up to 200 °C, held for 1 min. The mass spectrometry detector was configured to operate with ionization of 70 eV in scanning mode (SCAN), in the mass range of 25–500 m/z. The temperatures were fixed at 200 °C for the ionization source and 180 °C for the quadrupole. The interface with the mass detector was kept at 200 °C.

## Results and discussion

### Specificity and linearity

**Fig. 1:**
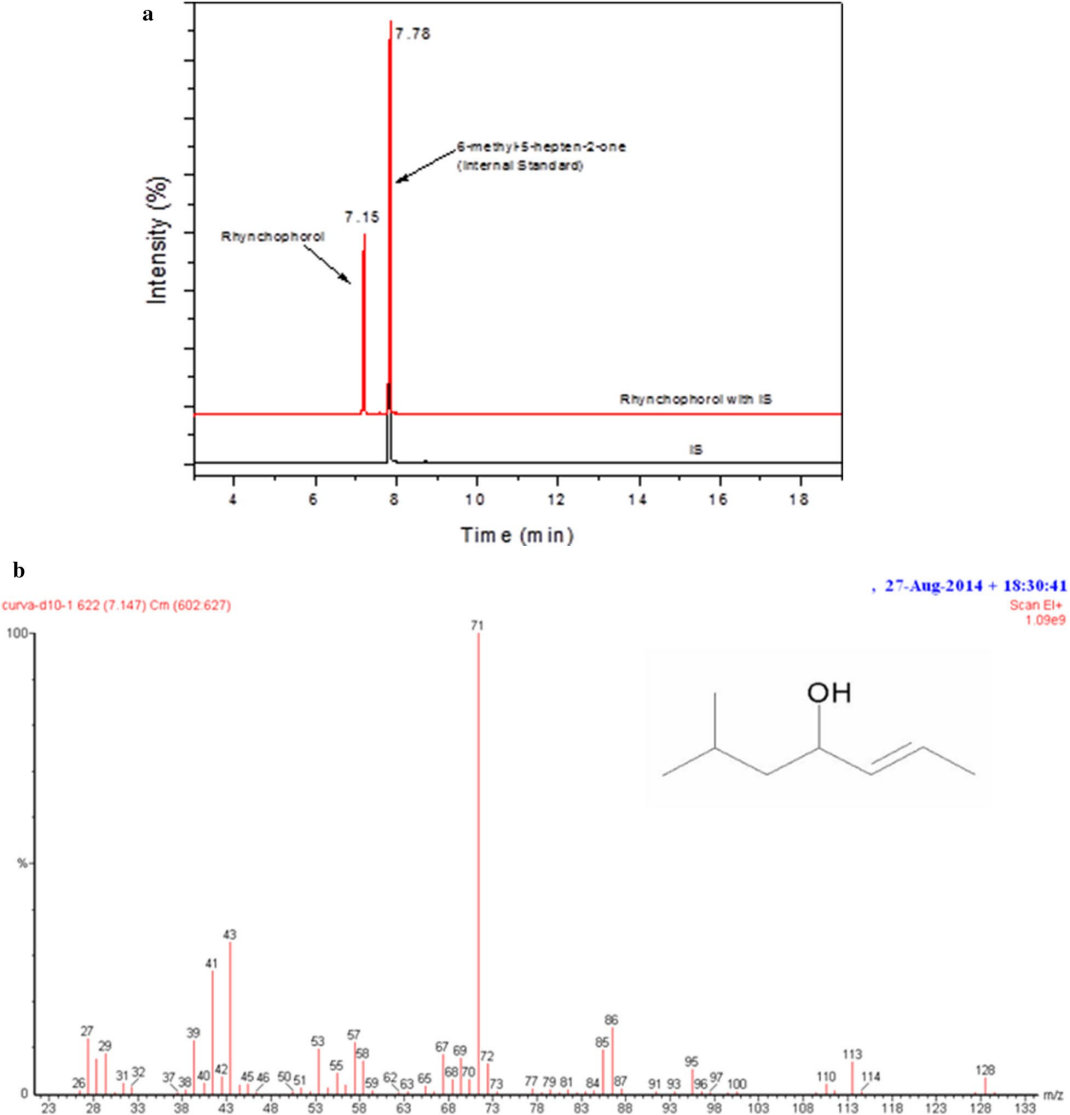
Chromatograms obtained under the analytical conditions of the method: **a** hexane solution containing rhynchophorol and IS; **b** mass spectrum obtained for rhynchophorol

The result obtained for the correlation coefficient was R^2^ = 0.9978, demonstrating good linearity for the calibration curve. This result is in compliance with the standard value required by ANVISA [16], which establishes acceptable linearity as an R^2^ value above 0.99 and an analytical curve of y = 0.062x + 0.1249 (Fig. 1).

The specificity of a method relates to its ability to accurately measure an analyte in the presence of other components that may be present in the sample, such as impurities, degradation products and other matrix components [18]. In this method, mass spectrometry was used for the detection of the pheromone. Ions characteristic of rhynchophorol were used for the identification: m/z (%) M^+^ 41 (2), 53 (13), 57 (12), 71 (100) and 128 (2)(19). The software program NIST Mass Spectral Search (version 2.0f), was used to aid the confirmation of the identification, and similarity above 80% was observed for rhynchophorol.

#### Precision, recovery, detection limit (DL) and quantification limit (QL)

The precision, considering the values for the coefficient of variance (CV%) and standard deviation (STD), obtained for the pheromone rhynchophorol are given in Table 1. The results show CV and STD values lower than 5%, satisfying the requirements established by ANVISA [16].

**Table 1:** Intermediary precision for the analytical method to determine the pheromone rhynchophorol

Concentration	Average	STD	CV%
1	0.0836	0.0013	1.59
20	1.2723	0.0040	0.31
50	2.4445	0.0165	0.68

The percentage recovery of the absorbed rhynchophorol from the composite (CR%) varied from 84 to 105%. These results are also in compliance the current legislation, which establishes recovery rates within the theoretical concentration range of 80 to 120%.

Values of 0.2 mg mL^−1^ for DL and 0.3 mg mL^−1^ for QL were obtained as the operational limits of the device used.

### Characterization of the synthesized Na-magadiite

**Fig. 2:**
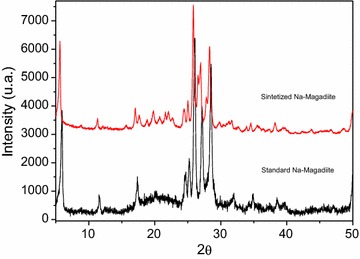
X-ray diffraction patterns for the synthesized and standard Na-magadiite

The Na-magadiite formation was confirmed through comparison of the XRD result with the standard provided by IZA (2017), as shown in Fig. 2.

Intensity peaks can be observed on the diffractogram for the angles characterizing the Na-magadiite formation: 5.62, 11.32, 17.06, 25.9, 26.96, 28.32, and 50.02.

In the EDX elemental analysis carried out on the synthesized Na-magadiite, a predominantly SiO_2_ (97.87% of the total composition) matrix was observed, with 1.89% of Al_2_O_3_. Trace levels of ZnO and CuO were also present.

### Rhynchophorol interaction with Na-magadiite and zeolite L

**Fig. 3:**
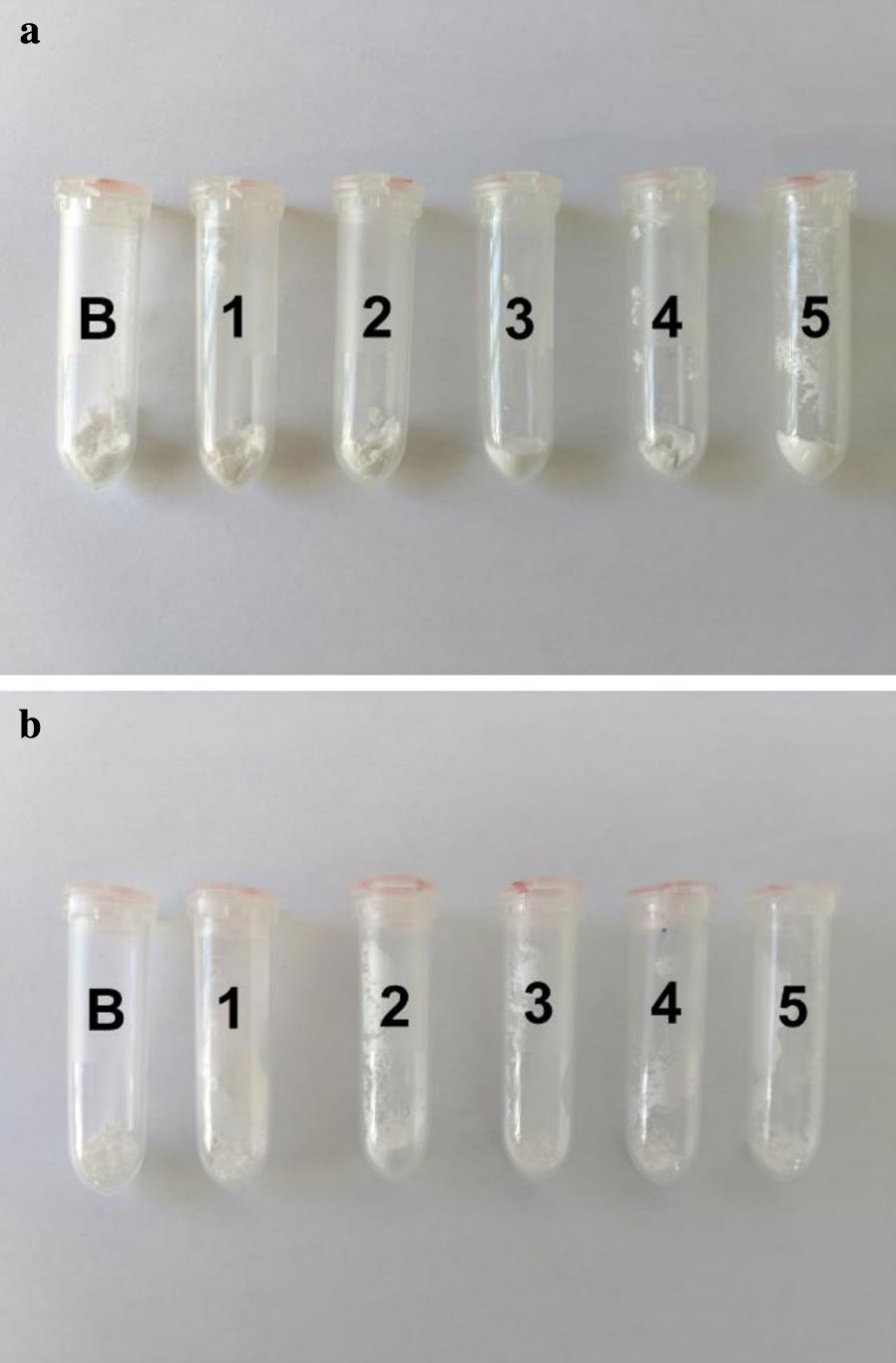
Matrices after a period of 24 h of rhynchophorol adsorption: **a** Na-magadiite; **b** zeolite L

According to Ramos et al. [7], one of the main reactions that demonstrates rhynchophorol degradation in the rhynchophorol + magadiite interaction is the color change of the material, which that can be seen with the naked eye. Figure 3 shows the rhynchophorol + magadiite and rhynchophorol + zeolite matrices after 24 h of interaction. It can be observed that the pheromone was not degraded in these interactions.

### Study on the controlled release of the rhynchophorol adsorbed on the magadiite and zeolite

**Fig. 4:**
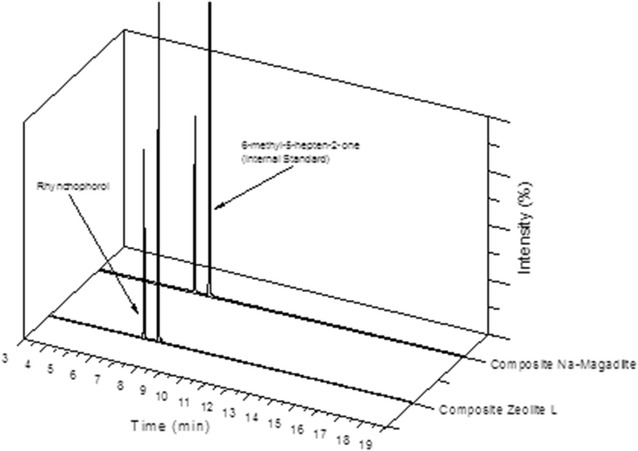
Chromatograms of the solutions recovered from the Na-magadiite and zeolite L composites

In order to confirm the presence of rhynchophorol in the composite formed with the Na-magadiite, the pheromone was recovered by extraction with *n*-hexane and quantified by the validated method. Typical chromatograms for the extracts obtained are given in Fig. 4.

It can be observed that the *n*-hexane solution obtained in the extraction process is similar to the standard of pure rhynchophorol. After 24 h of adsorption and the subsequent extraction, it was possible to recover 89.05% of rhynchophorol, which highlights the protection of this pheromone in the matrix studied. It was also observed that the formation of new peaks did not occur, indicating that rhynchophorol degradation products were not generated. In a study carried out by Ramos et al. [7], zeolites with an MFI spatial conformation (ZSM-5 and silicalite-1) were used as a device to for the controlled release of rhynchophorol and it was verified that the characteristics of the adsorbent matrix are essential factors in avoiding the pheromone degradation during the adsorption process. Structures with high AI ratios in the network formation promote higher Lewis acidity and an increase in the diameter of the channels, facilitating the access of pheromone to the interior of the structure, leading to greater degradation of the pheromone studied.

**Fig. 5:**
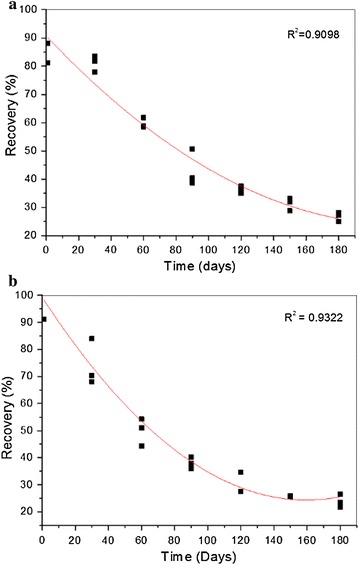
Long-term stability test for rhynchophorol adsorbed on Na-magadiite (**a**) and zeolite L (**b**)

Figure 5 shows the values for the rhynchophorol adsorbed on Na-magadiite as a function of the storage time, simulating the stability condition at ambient temperature.

The rhynchophorol adsorbed on the matrix shows an exponential mass loss behavior during storage for 180 days (Fig. 5). The pheromone release rate was 0.89 ± 0.41 mg day^−1^ in the first 30 days, due to the dispersion of the pheromone in the matrix. After 30 days, the release rate decreased to approximately 0.046 ± 0.008 mg day^−1^, with the controlled release of the pheromone being observed throughout the period evaluated. The same behavior was noted for the composite formed with zeolite L, which showed a release rate of 0.517 ± 0.68 mg day^−1^ in the first 30 days, reducing to an average rate of 0.0539 ± 0.0154 mg day^−1^ for the remainder of the period.

Vacas et al. [19] used the aggregation pheromone ferruginol in its liquid form to capture *R. ferrugineus Olivier*. It was placed in LDPE vials to simulate the constant release rates in the traps. The authors noted that the release of this pheromone at a rate of 2.6 mg day^−1^ is sufficient to attract the pest. However, lower release rates can be achieved when the pheromone is adsorbed onto a matrix, which promotes its slower release into the environment over a longer period of time.

Stipanovic et al. [20] carried out controlled release tests on the pheromone codlemone adsorbed on cellulose derivatives surrounded by a polymeric membrane, aimed at its application in the control of Lepidopteran pests (moths). They obtained release rates of around 0.784 mg day^−1^, which was similar to the value obtained in this study for the composite formed with the Na-magadiite. Since zeolite L is a three-dimensional network of channels, the release of the rhynchophorol adsorbed on this matrix was slower. This was also observed by Ramos et al. [7] for the zeolite silicalite-1. Release rates for the pheromone rhynchophorol varying from 0.002592 to 0.2592 mg day^−1^ are favorable for the identification and the attraction of *R. palmarum* L., showing that the two matrices used in this study have the potential for application together with traps for periods of up to 180 days [21].

**Fig. 6:**
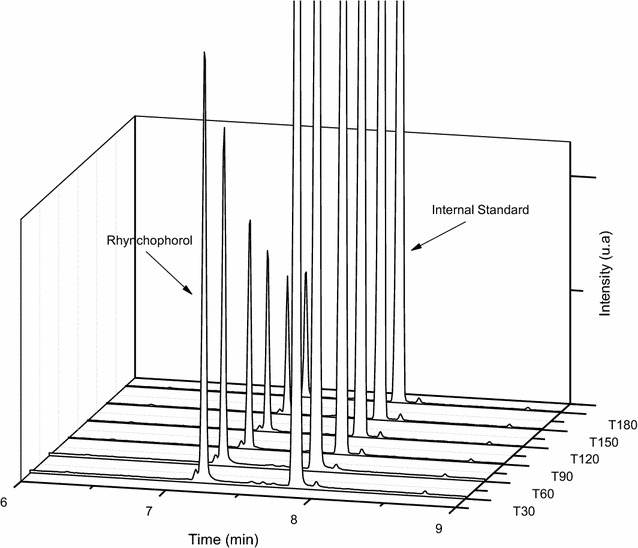
Storage of Na-magadiite/rhynchophorol composite for intervals of up to 180 days

In order to evaluate the stability of the rhynchophorol adsorbed on the Na-magadiite during its long-term storage, the composite was evaluated considering the possibility of degradation with the formation of new compounds. In Fig. 6, the maintenance of rhynchophorol (tr = 7.18 min) and the IS (tr = 7.84 min) can be observed.

During the storage period, new peaks were not observed in the analysis, confirming that the reduction in the rhynchophorol values for the studied matrices was due to release and not to the degradation of this pheromone (Fig. 6). Ramos et al. [2, 7] observed that pure silica zeolites, of the type silicalite-1, were also able to store rhynchophorol for long periods without its degradation. In contrast, in the case of zeolite ZSM-5, when used for the same purpose, pheromone degradation was observed within less than 30 days of storage. The cited authors attributed the degradation to acids in the matrix and diffusion within the structure, leading to access to free Brønsted acid sites.

## Conclusions

In this study, stable matrices of Na-magadiite and zeolite L containing rhynchophorol were successfully prepared. The analytical methodology for the determination of rhynchophorol was considered adequate with regard to the proposed application, since it showed good values for recovery, linearity, DL and QL. The characterization of the matrix highlights that rhynchophorol remained stable and did not degrade on interaction with the inorganic matrix. The study confirmed that the controlled release of the pheromone occurred at rates that enable the identification and the attraction of the target insect. It was possible to obtain a stable complex for the controlled release of the pheromone, which could be used in the future for the control of *Rhynchophorus palmarum* L., insects that can cause the destruction of cultures such as coconut trees and oil palm trees. This approach can be applied in the form of tablets or in plastic Eppendorf^®^ Safe-Lock tubes or similar materials, as described in the patent request BR1020150326041, registered at the National Institute for Industrial Property—INPI/BR.

## Highlights

- Fast and low cost analytical method for the quantification and stability evaluation of the pheromone rhynchophorol;
- Analytical method for the adsorption and recovery of pheromone using inorganic matrices;
- Elaboration of an inorganic matrix/pheromone composite aimed at pest control through controlled pheromone release.
